# Development and validation of a prediction model based on machine learning algorithms for predicting the risk of heart failure in middle‐aged and older US people with prediabetes or diabetes

**DOI:** 10.1002/clc.24104

**Published:** 2023-07-31

**Authors:** Yicheng Wang, Riting Hou, Binghang Ni, Yu Jiang, Yan Zhang

**Affiliations:** ^1^ Department of Cardiovascular medicine Affiliated Fuzhou First Hospital of Fujian Medical University Fuzhou Fujian China; ^2^ The Third Clinical Medical College Fujian Medical University Fuzhou Fujian China; ^3^ Cardiovascular Disease Research Institute of Fuzhou City Fuzhou Fujian China

**Keywords:** diabetes, heart failure, machine learning, national health and nutrition survey, NHANES, prediabetes, prediction model

## Abstract

**Background:**

The purpose of this study was to develop and validate a machine learning (ML) based prediction model for the risk of heart failure (HF) in patients with prediabetes or diabetes.

**Methods:**

We used 3527 subjects aged 40 years and older with a prior diagnosis of prediabetes or diabetes from the National Health and Nutrition Examination Survey (NHANES) from 2007 to 2018. The search for independent risk variables linked to HF was conducted using univariate and multivariable logistic regression analysis. The 3527 subjects were randomly divided into training set and validation set in a 7:3 ratio. Five ML models were built on the training set using five ML algorithms, including random forest (RF), and then validated on the validation set. Receiver operating characteristic (ROC) curves, calibration curves, and decision curve analysis and Bootstrap resampling method were used to measure the predictive performance of the five ML models.

**Results:**

Multivariate logistic regression analysis showed that age, poverty‐to‐income ratio, myocardial infarction condition, coronary heart disease condition, chest pain condition, and glucose‐lowering medication use were independent predictors of HF. By comparing the performance of the five ML models, the RF model (AUC = 0.978) was the best prediction model.

**Conclusions:**

The risk of HF in middle‐aged and elderly patients with prediabetes or diabetes can be accurately predicted using ML models. The best prediction performance is presented by RF model, which can assist doctors in making clinical decisions.

## Graphical abstract

The performance of five machine learning models was compared using receiver operating characteristic curves, calibration curves, and decision curve analysis, and the random forest model was found to be the best model for predicting the risk of heart failure in a prediabetes or diabetes population.

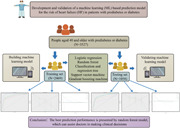

## 1 INTRODUCTION

Heart failure (HF) has grown significantly in importance as a public health issue and as a population health danger in recent decades.^1^ According to general estimates, 1−2% of the adult population in developed nations are known to have HF.^2^, ^3^ HF also has a high hospitalization rate and places a heavy socioeconomic strain on society.^4^, ^5^

Diabetes is one of an independent risk factor for HF, which has been confirmed in many studies.^6^, ^7^, ^8^ It significantly contributes to the development of HF by directly impairing cardiac function and indirectly doing so through diseases including hypertension, heart disease, renal insufficiency, obesity, and other metabolic conditions.^9^ It is crucial to successfully prevent and treat two concurrent diseases because patients' prognoses are also poorer when they have two diseases.^10^

Prediabetes, which includes impaired fasting glucose and impaired glucose tolerance (IGT), is the metabolic condition in between diabetes and normoglycemia.^11^, ^12^ According to the American Diabetes Association, the current definition of prediabetes in the United States includes fasting blood glucose of 100 to 125 mg/dL, postload plasma glucose of 140 to 199 mg/dl, or HbA1c of 5.7% to 6.4%.^13^ Several studies have confirmed that prediabetes is associated with poor outcomes in HF and may further increase the risk when developing diabetes mellitus.^14^, ^15^, ^16^, ^17^

Currently, artificial intelligence is widely used in the medical field, and machine learning (ML) is increasingly used in medical treatment, prediction and diagnosis of diseases.^18^, ^19^, ^20^, ^21^, ^22^, ^23^, ^24^ The use of ML algorithms in medicine can result in more precise illness diagnosis and personalized treatment for individualized patients.^25^, ^26^ In comparison to conventional statistical methods, ML algorithms have better predictive performance, according to research on predictive models based on these algorithms.^27^

Early and accurate detection of the risk of HF is essential to reducing the related adverse health effects given the connection between diabetes or prediabetes and HF. There are few predictive models for the risk of pre‐diabetes or diabetic HF constructed using ML algorithms. This study used a ML approach to develop a predictive model for HF based on data from the National Health and Nutrition Examination Survey (NHANES) from 2007 to 2018 to identify at‐risk populations. The development of a prediction model based on the risk of developing HF in patients with prediabetes or diabetes could help to accurately identify individuals at risk for HF death in patients with diabetes, providing an opportunity to personalize preventive therapy and potentially reduce the burden of cardiovascular disease in patients with prediabetes or diabetes.

## 2 METHODS

### 2.1 Study populations

**Figure 1:**
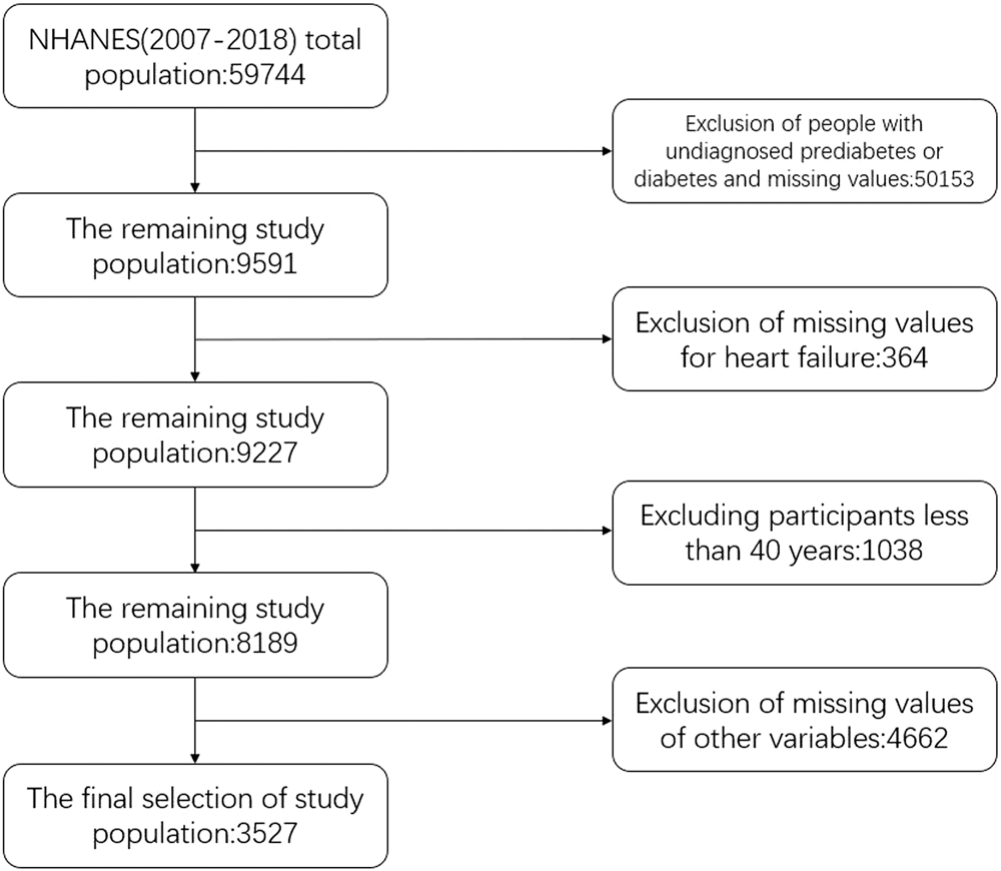
The flowchart of participants.

In this study, we used data sets from the NHANES from 2007 to 2018 for training and internal validation. The NHANES program use a sophisticated multistage sampling design to collect an annual sample of roughly 5000 people who are nationally representative, and it refreshes the database every 2 years. We extracted data from a total of 59 744 participants in NHANES from 2007 to 2018. Exclusion criteria were: (1) Participants with no previous diagnosis of prediabetes or diabetes; (2) People under 40 years of age; (3) Of all variables included by participants in this study, at least one variable had a missing value. In the end, we selected 3527 participants with a history of prediabetes or diabetes who were 40 years of age and older. The NCHS Institutional Review Board gave their approval to the survey protocol for the gathering of NHANES data. The screening flow chart of the participant population is shown in Figure 1. Study protocols for NHANES were approved by the NCHS ethnics review board (Protocol #2011–17, https://www.cdc.gov/nchs/nhanes/irba98.htm). All study participants, in the NHANES data we utilized, provided informed consent before their participation in the NHANES survey, as per the NHANES protocol.

### 2.2 Data collection

As part of the questionnaire in NHANES. People 40 years of age and older who had ever been told by a doctor that they had diabetes or prediabetes were selected to participate in the survey. Prediabetes was asked “Have you ever been told by a doctor or other health care provider that you have prediabetes, impaired fasting blood sugar, IGT, borderline diabetes, or that your blood sugar is higher than normal but not high enough to be classified as diabetes or sugar diabetes?” If the subject answered “yes,” he or she was considered to be Prediabetes. Within NHANES there is a question “have you ever been told by a doctor or health professional that you have diabetes?” For our study, an affirmative answer to this question was used to classify patients as having diabetes. Similarly, Subjects' HF was also collected through the NHANES questionnaire format. There is a question “have you ever been diagnosed with HF by a doctor.” If the subject answered “yes” then the subject was considered in our study to have HF.

Within the NHANES study, a self‐reported questionnaire was used to gather patient demographic information. Demographic characteristics were categorized by gender (male, female), race (non‐Hispanic white, non‐Hispanic black, Mexican American, Hispanic American, other race), marital status (unmarried, married or living with partner, married but currently living alone [separated, divorced, or widowed]), and education level (less than 9th grade, 9th−11th grade, high school graduate, partial college or AA graduate or above). As part of their self‐reported medical history, participants were asked if they had ever been told they had coronary heart disease, chest pain, myocardial infarction, hypertension. There are three medication status: not taking a glucose‐lowering drug, taking a glucose‐lowering drug, and taking other prescribed medications but not a glucose‐lowering drug. Information on smoking and alcohol consumption was also included in the questionnaire section. The smoking status of each participant was assessed by self‐report, and they were divided into three groups: nonsmokers, former smokers, and current smokers. Within the NHANES database, drinking status is categorized as never drinking, former drinkers now abstaining, heavy drinkers (≥3 drinks per day for women/≥4 drinks per day for men/five or more days of binge drinking per puff), moderate drinkers (≥2 drinks per day for women/≥3 drinks per day for men/≥2 days of binge drinking per puff), and light drinkers (excluding the above). Waist circumference (WC), body mass index (BMI) was obtained by medical staff at mobile screening stations. BMI is calculated by dividing weight in kilograms by the square of height in meters (kg/m^2^). Mean systolic and mean diastolic blood pressures were the average of four blood pressures measured in subjects at the time of screening. The individuals were questioned, “How much time do you typically sit in a day?” to determine the amount of sedentary time. The sleep time on workdays was determined by asking participants, “How much sleep do you typically receive on a workday?” Physical activity time was obtained by asking the subjects “how long they exercised in a week.” The household (or individual) income was divided by the survey year and the state‐specific poverty line to determine the poverty‐to‐income ratio (PIR). Estimated glomerular filtration rate (eGFR) was calculated by the Chronic Kidney Disease Epidemiology Consortium formula.^28^ Some other blood specimens such as uric acid (UA), creatinine (CR), blood urea nitrogen (BUN), albumin (ALB), triglycerides, total cholesterol (TCHOL), high density lipoprotein and HbA1c are measured from the laboratory.

### 2.3 Development and validation of prediction models

By using weighted univariate and multivariate logistic regression, our study identified separate risk factors for the risk of HF in individuals with prediabetes or diabetes. Following that, ML prediction models were created using the variables chosen by stepwise regression as input variables. The ratio of 7:3 was used to randomly divide all participants into a training set for building the prediction model and a validation set for validating the model. On the training set, models were built using five ML algorithms, including logistic regression (LR), random forest (RF), classification and regression tree (CART), gradient boosting machine (GBM), and support vector machine (SVM). The performance of each model is validated and compared in the validation set. The receiver operating characteristic (ROC) curve of the five ML models were plotted in the training and validation sets, and the area under the ROC curve (AUC) were calculated to evaluate the predictive power of the five ML models. The model with the largest AUC was selected as the best model for each algorithm. Calibration curve was used to compare the degree of calibration of each model. Decision curve analysis (DCA) was used to analyze and compare them for clinical applications. To confirm whether the model is overfitting, internal model validation is performed using Bootstrap method.

### 2.4 Statistical analysis

Considering the characteristics of the complex sampling design of NHANES, we used weighting in the baseline description, univariate logistic regression analysis, and multivariate logistic regression analysis. Categorical variables are expressed as numbers (*n*) and percentages (%) while continuous variables are expressed as means and standard errors. The chi‐square test or Fisher's exact test was employed to ascertain between‐group differences for categorical variables. To identify statistical differences in the proportions of the groups, weighted one‐way ANOVA was performed to calculate odds ratios (OR) and 95% confidence intervals. *p* < .05 indicates that the difference is statistically significant. All analyses were performed with R (version 4.2.3).

## 3 RESULTS

### 3.1 Baseline characteristics

3527 participants in total had previously been identified as having prediabetes or diabetes after inclusion and exclusion. Table 1 displays the clinical traits of the weighted study population. Of these 3527 subjects, the mean age was 59.62 years, with 2035 men and 1492 women. 40.57% were Non‐Hispanic white, 21.49% were Non‐Hispanic black, 16.36% were Mexican‐American, 10.77% were Hispanic American, and 10.8% were of other races.

**Table 1:** Weighted baseline characteristics.

Variables	Total	Training no	Set yes	*p* Value	Testing no	Setyes	*p* Value
*N*	3527	2330	139		1008	50	
AGE (years)	59.62 (0.28)	59.29 (0.32)	67.50 (0.94)	<.001	59.07 (0.46)	67.96 (1.26)	<.001
BMI (kg/m²)	31.30 (0.17)	31.04 (0.21)	34.54 (1.12)	.003	31.47 (0.29)	31.64 (1.43)	.91
WC (cm)	107.36 (0.36)	106.36 (0.46)	115.24 (1.70)	<.001	108.51 (0.72)	109.27 (3.86)	.85
PIR	3.18 (0.05)	3.20 (0.05)	2.46 (0.20)	<.001	3.24 (0.09)	2.51 (0.35)	.06
Sleep (h)	7.17 (0.04)	7.14 (0.04)	7.39 (0.18)	.18	7.19 (0.07)	7.00 (0.25)	.46
Sedentary (min)	364.80 (4.63)	355.33 (5.75)	415.11 (18.28)	.003	378.12 (9.22)	391.65 (24.38)	.6
Exercise (min)	483.50 (17.51)	501.84 (22.51)	341.29 (42.35)	.002	462.06 (28.14)	447.50 (146.32)	.92
UA (mg/dL)	5.77 (0.03)	5.68 (0.04)	6.43 (0.19)	<.001	5.88 (0.07)	5.88 (0.34)	.99
CR (mg/dL)	0.93 (0.01)	0.92 (0.01)	1.20 (0.07)	<.001	0.93 (0.02)	0.99 (0.05)	.19
BUN (mg/dL)	15.18 (0.12)	14.90 (0.17)	19.85 (0.72)	<.001	15.15 (0.23)	17.54 (0.96)	.02
ALB (mg/dL)	4.21 (0.01)	4.22 (0.01)	4.08 (0.04)	<.001	4.22 (0.02)	4.14 (0.05)	.12
TG (mg/dL)	180.13 (4.24)	183.58 (5.60)	183.21 (9.50)	.97	172.71 (4.97)	174.35 (19.98)	.94
TCHOL (mg/dL)	192.74 (1.28)	194.55 (1.57)	181.99 (6.29)	.05	190.83 (2.03)	175.36 (5.83)	.02
eGFR (mL/min)	84.33 (0.46)	84.80 (0.52)	68.65 (2.79)	<.001	85.40 (0.78)	77.14 (3.02)	.01
HDL (mg/dL)	49.77 (0.39)	50.30 (0.47)	47.09 (1.71)	.08	48.94 (0.66)	49.99 (2.57)	.69
SYS (mmHg)	128.50 (0.40)	128.44 (0.43)	127.18 (1.73)	.5	128.66 (0.84)	130.77 (2.55)	.44
DIA (mmHg)	71.09 (0.30)	71.44 (0.34)	64.98 (1.58)	<.001	71.16 (0.65)	67.69 (2.29)	.16
HbA1c (%)	6.49 (0.03)	6.47 (0.04)	6.75 (0.15)	.08	6.50 (0.05)	6.94 (0.33)	.19
Education				.05			<.001
Less than 9th grade	442 (12.53%)	299 (6.21%)	16 (6.49%)		118 (4.60%)	9 (12.48%)	
9‐11th grade	489 (13.86%)	322 (10.40%)	19 (12.34%)		141 (9.77%)	7 (7.68%)	
High school graduate	822 (23.31%)	539 (24.51%)	43 (30.85%)		230 (24.95%)	10 (26.27%)	
Some college graduate	1008 (28.58%)	651 (30.47%)	47 (38.10%)		290 (29.78%)	20 (49.12%)	
College graduate or above	766 (21.72%)	519 (28.42%)	14 (12.23%)		229 (30.89%)	4 (4.45%)	
Marital				.02			.37
Never married	277 (7.85%)	190 (6.93%)	12 (8.68%)		73 (7.06%)	2 (2.64%)	
Living with partner	2257 (63.99%)	1490 (69.21%)	71 (52.93%)		667 (71.01%)	29 (67.61%)	
Widowed/divorced	993 (28.15%)	650 (23.86%)	56 (38.39%)		268 (21.93%)	19 (29.75%)	
Race				.01			.47
Non‐Hispanic White	1431 (40.57%)	913 (67.81%)	67 (73.70%)		423 (72.65%)	28 (68.68%)	
Non‐Hispanic Black	758 (21.49%)	507 (10.99%)	39 (14.85%)		203 (9.26%)	9 (9.57%)	
Mexican American	577 (16.36%)	379 (7.88%)	12 (4.52%)		182 (7.85%)	4 (3.86%)	
Other Hispanic	380 (10.77%)	259 (4.93%)	13 (3.86%)		102 (3.97%)	6 (5.11%)	
Other Race	381 (10.8%)	272 (8.39%)	8 (3.07%)		98 (6.27%)	3 (12.79%)	
Sex				.76			.53
Female	1492 (42.3%)	1014 (43.69%)	53 (45.81%)		409 (40.19%)	16 (33.72%)	
Male	2035 (57.7%)	1316 (56.31%)	86 (54.19%)		599 (59.81%)	34 (66.28%)	
Smoke				.02			.07
Never	1785 (50.61%)	1193 (50.32%)	56 (39.37%)		517 (50.89%)	19 (34.25%)	
Former	1204 (34.14%)	771 (35.51%)	58 (48.67%)		354 (36.72%)	21 (38.24%)	
Now	538 (15.25%)	366 (14.17%)	25 (11.95%)		137 (12.40%)	10 (27.52%)	
Alcohol				.8			.03
Never	542 (15.37%)	376 (12.03%)	19 (11.76%)		141 (12.55%)	6 (10.89%)	
Former	739 (20.95%)	476 (16.77%)	30 (17.95%)		217 (17.38%)	16 (37.36%)	
Mild	1348 (38.22%)	898 (44.85%)	59 (49.12%)		371 (39.84%)	20 (35.42%)	
Moderate	417 (11.82%)	274 (13.09%)	14 (12.18%)		124 (15.48%)	5 (8.71%)	
Heavy	481 (13.64%)	306 (13.26%)	17 (8.99%)		155 (14.75%)	3 (7.62%)	
MI				<.001			<.001
No	3256 (92.32%)	2208 (95.31%)	75 (59.52%)		947 (93.86%)	26 (62.45%)	
Yes	271 (7.68%)	122 (4.69%)	64 (40.48%)		61 (6.14%)	24 (37.55%)	
Chest pain				<.001			<.001
No	3338 (94.64%)	2241 (96.40%)	98 (73.17%)		967 (96.26%)	32 (63.07%)	
Yes	189 (5.36%)	89 (3.60%)	41 (26.83%)		41 (3.74%)	18 (36.93%)	
Hypertension				.001			.004
No	811 (22.99%)	543 (24.65%)	11 (8.06%)		251 (25.83%)	6 (7.87%)	
Yes	2716 (77.01%)	1787 (75.35%)	128 (91.94%)		757 (74.17%)	44 (92.13%)	
CHD				<.001			<.001
No	3225 (91.44%)	2191 (93.88%)	66 (48.60%)		939 (92.49%)	29 (69.06%)	
Yes	302 (8.56%)	139 (6.12%)	73 (51.40%)		69 (7.51%)	21 (30.94%)	
Take drug for diabetes				<.001			.04
No	648 (18.37%)	443 (18.52%)	2 (1.15%)		201 (16.57%)	2 (2.01%)	
Yes	1502 (42.59%)	975 (36.98%)	82 (60.21%)		420 (40.14%)	25 (49.13%)	
Other	1377 (39.04%)	912 (44.49%)	55 (38.63%)		387 (43.29%)	23 (48.86%)	

Abbreviations: ALB, albumin; BMI, body mass index; BUN, blood urea nitrogen; CHD, coronary heart disease; CR, creatinine; DIA, diastolic; eGFR, estimated glomerular filtration rate; HDL, high density lipoprotein; MI, myocardial infarction; PIR, poverty‐to‐income ratio; SYS, systolic; TCHOL, total cholesterol; TG, triglyceride; UA, uric acid; WC, waist circumference.

The total population consisted of 2469 individuals in the training set and 1058 individuals in the validation set. The training and validation sets were divided into two groups according to the presence or absence of HF. The training set was statistically significant in age, race, education, marital status, smoking status, BMI, WC, PIR, sedentary time, physical activity time, serum UA, serum CR, serum urea nitrogen, serum ALB, TCHOL, eGFR, diastolic blood pressure, coronary heart disease condition, chest pain condition, myocardial infarction condition, hypertension condition, and taking glucose‐lowering medication condition (*p* < .05). The validation set was statistically significant in age, education, alcohol consumption, urea nitrogen, TCHOL, eGFR, coronary heart disease condition, chest pain condition, myocardial infarction condition, hypertension condition, and use of hypoglycemic drugs condition (*p* < .05).

### 3.2 Univariate and multivariate logistic regression analysis of HF

To identify variables associated with the risk of HF in patients with prediabetes or diabetes, we analyzed 29 variables. We performed successively univariate and multivariate logistic regression analyses to explore independent risk factors for HF. In the univariate analysis, age, education, smoking status, BMI, PIR, WC, UA, CR, BUN, ALB, TCHOL, diastolic blood pressure, sedentary time, eGFR, HbA1c, coronary heart disease, chest pain, hypertension, myocardial infarction, and glucose‐lowering medication were significantly associated with the development of HF (*p* < .05). Multivariate logistic regression showed that age, PIR, myocardial infarction condition, coronary heart disease condition, chest pain condition, and glucose‐lowering medication condition were independent risk factors for HF. The results of weighted univariate and multivariate logistic analysis are shown in Table 2.

**Table 2:** Weighted univariate and multivariate logistic regression analysis.

Variables	Univariate OR (95% CI)	*p* Value	Multivariate OR (95% CI)	*p* Value
AGE (years)	1.07 (1.06−1.09)	<.001	1.04 (1.01−1.07)	.010
BMI (kg/m²)	1.05 (1.02−1.09)	.002	/	/
WC (cm)	1.03 (1.01−1.04)	<.001	/	/
PIR	0.76 (0.66−0.87)	<.001	0.74 (0.58−0.95)	.020
Sleep (h)	1.07 (0.90−1.26)	.45	/	/
Sedentary (min)	1.00 (1.00−1.00)	.01	/	/
Exercise (min)	1.00 (1.00−1.00)	.12	/	/
UA (mg/dL)	1.27 (1.11−1.44)	<.001	/	/
CR (mg/dL)	1.59 (1.12−2.24)	.01	/	/
BUN (mg/dL)	1.07 (1.06−1.10)	<.001	/	/
ALB (mg/dL)	0.32 (0.18−0.56)	<.001	/	/
TG (mg/dL)	1.00 (1.00−1.00)	.94	/	/
TCHOL (mg/dL)	0.99 (0.99−1.00)	.02	/	/
HDL (mg/dL)	0.99 (0.98−1.01)	.21	/	/
SYS (mmHg)	1.00 (0.99−1.01)	.83	/	/
DIA (mmHg)	0.97 (0.95−0.98)	<.001	/	/
eGFR (mL/min)	0.97 (0.96−0.98)	<.001	/	/
HbA1c (%)	1.14 (1.03−1.27)	.02	/	/
Education				
Less than 9th grade	Ref.	Ref.	Ref.	Ref.
9‐11th grade	0.76 (0.37−1.57)	.45	/	/
High school graduate	0.84 (0.44−1.62)	.60	/	/
Some college graduate	0.95 (0.52−1.74)	.87	/	/
College graduate or above	0.24 (0.10−0.58)	.002	/	/
Marital				
Never married	Ref.	Ref.	Ref.	Ref.
Living with Partner	0.81 (0.31−2.14)	.67	/	/
Widowed/Divorced	1.54 (0.60−3.93)	.37	/	/
Race				
Non‐Hispanic White	Ref.	Ref.	Ref.	Ref.
Non‐Hispanic Black	1.23 (0.84−1.82)	.29	/	/
Mexican American	0.53 (0.27−1.04)	.06	/	/
Other Hispanic	0.87 (0.51−1.48)	.61	/	/
Other race	0.71 (0.26−1.98)	.51	/	/
Sex				
Female	Ref.	Ref.	Ref.	Ref.
Male	1.00 (0.63−1.60)	.99	/	/
Smoke				
Never	Ref.	Ref.	Ref.	Ref.
Former	1.70 (1.17,2.46)	.01	/	/
Now	1.58 (0.90−2.80)	.11	/	/
Alcohol				
Never	Ref.	Ref.	Ref.	Ref.
Former	1.45 (0.83−2.54)	.19	/	/
Mild	1.11 (0.65−1.89)	.70	/	/
Moderate	0.86 (0.38−1.92)	.71	/	/
Heavy	0.66 (0.33−1.33)	.20	/	/
MI				
No	Ref.	Ref.	Ref.	Ref.
Yes	12.10 (7.58−19.31)	<.001	3.17 (1.48−6.82)	.004
Chest pain				
No	Ref.	Ref.	Ref.	Ref.
Yes	11.10 (7.07−17.43)	<.001	2.46 (1.28−4.72)	.008
CHD				
No	Ref.	Ref.	Ref.	Ref.
Yes	12.04 (8.02−18.09)	<.001	3.51 (1.82−6.75)	<.001
Hypertension				
No	Ref.	Ref.	Ref.	Ref.
Yes	3.83 (2.02−7.26)	<.001	/	/
Take drugs for diabetes				
No	Ref.	Ref.	Ref.	Ref.
Yes	19.43 (7.01−53.86)	<.001	4.77 (1.54−14.77)	.007
Other	12.13 (4.40−33.43)	<.001	4.41 (1.49−13.10)	.008

Abbreviations: ALB, albumin; BMI, body mass index; BUN, blood urea nitrogen; CHD, coronary heart disease; CR, creatinine; DIA, diastolic; eGFR, estimated glomerular filtration rate; HDL, high density lipoprotein; MI, myocardial infarction; PIR, poverty‐to‐income ratio; SYS, systolic; TCHOL, total cholesterol; TG, triglyceride; UA, uric acid; WC, waist circumference.

### 3.3 Predictive performance of ML algorithms

Based on six independent risk factors for HF identified by multivariate logistic regression analysis in the training set, we used five different ML algorithms, including CART, RF, SVM, GBM, and logistic regression, to construct five prediction models for HF risk in prediabetes or diabetes. The predictive performance of five ML models was evaluated in the training set using ROC curve, with the RF model performing best in predicting the risk of HF in the prediabetes or diabetes population (AUC = 0.978), followed by the GBM model (AUC = 0.873), logistic regression model (AUC = 0.870), SVM model (AUC = 0.837), and CART (AUC = 0.822) model (Supporting Information: Figure S1). Similarly, the RF (AUC = 0.865) model also showed the best performance among the ROC curves of the five ML models identified on the validation set by internal validation (Supporting Information: Figure S2). The calibration curve of the training set shows that the predictive power of the RF model is very similar to the actual results (Supporting Information: Figure S3). Of course, the calibration curve of the validation set shows that the RF model also performs well (Supporting Information: Figure S4). The DCA curve from the training set illustrates that among the five ML models, the RF performs the best, confirming its good clinical application (Supporting Information: Figure S5). The DCA curve from the validation set shows that the RF model has a significant positive net benefit in predicting risk (Supporting Information: Figure S6). The model was then internally validated using the Bootstrap method with an internal validation AUC of 0.834. Therefore, the RF model was finally selected as the prediction model in this study.

### 3.4 Influence of variables on prediction performance

The relative importance ranking of the six risk factors varied among the five ML models. In the RF model, the relative importance of each variable in descending order was PIR, age, myocardial infarction condition, coronary heart disease condition, chest pain condition, and taking glucose‐lowering medication condition. The results are shown in Supporting Information: Figure S7.

## 4 DISCUSSION

In this study, age, PIR, myocardial infarction condition, coronary heart disease condition, chest pain condition, and glucose‐lowering medication use were screened as predictors of HF by univariate and multivariate logistic regression analysis.

Based on these predictors, five ML algorithms were developed and validated using five ML models to predict the risk of HF in patients with prediabetes or diabetes. By comparing the degree of calibration, discrimination, and clinical utility of the five ML models, the results showed that the RF model had the best predictive ability for HF in patients with prediabetes or diabetes.

The PIR ranked highest among the five predictors in the RF model. According to our data results, the risk of HF was negatively correlated with the PIR. The effect of income on HF has been confirmed in many studies, with low‐income groups being more likely to develop HF or have a higher mortality rate from HF than higher‐income groups, possibly because low‐income groups tend to receive more limited medical resources.^29^, ^30^, ^31^

Age was the second most important predictor. The expected 5‐year mortality from HF in diabetic people younger than 75 years of age increased dramatically with age, according to the findings of a prospective research involving 283 patients with chronic HF.^32^ A person personal sensitivity to HF and risk of developing HF rises with increasing age due to changes in the structure and function of the heart.^33^ Consequently, it is important to increase the screening for cardiovascular disease in older people to reduce the risk of the condition and start targeted treatment early.

As a cardiovascular disease with an acute onset, a large proportion of myocardial infarction survivors will eventually develop HF.^34^ In addition to this, a Taiwan‐based study found that certain coronary conditions, including percutaneous intracavitary coronary angiography, coronary artery bypass surgery and acute myocardial infarction, largely exacerbate the risk of HF in patients with diabetes.^35^ Chest pain is one of the primary symptoms of coronary heart disease, which has been demonstrated to be the primary cause of HF.^36^ As a result, there is a pressing need to further the study of coronary heart disease treatment and to identify the most appropriate treatment for individual patients through several studies.

In our study, people with diabetes who took glucose‐lowering drugs had a significantly lower risk of HF compared to patients who did not take glucose‐lowering drugs. Several studies have demonstrated that patients with HF benefit greatly from glucose‐lowering medications such dapagliflozin, canagliflozin, and empagliflozin by reducing mortality and improving outcomes.^37^, ^38^, ^39^, ^40^, ^41^ Several current anti‐glycemic medications have different effects on HF.^42^ Unfortunately, we extracted the use of hypoglycemic drugs, but based on the limitations of the NHANES database, we do not know the specific names of the drugs used in different subjects. To further understand the precise mechanisms of action of different glucose‐lowering medications on HF, more experimental studies are still required.

RF algorithm is an accurate, easy to interpret and computationally efficient ML algorithm defined by a collection of learning methods for classification, regression and other tasks.^43^ After verifying that the RF model exhibits excellence and outperforms the five ML methods in this study. In addition, ML models are likely susceptible to the problem of model overfitting.^44^ And yet, our results show that the internal validation by Bootstrap method has an AUC of 0.834. The AUC of the RF model is 0.978 on the training set and 0.865 on the test set, and the difference between the two results is not significant, indicating that our model is not severely overfitted.

There are few studies on using ML methods to predict the risk of HF in people with diabetes or prediabetes. To our knowledge, this is the first study to develop a predictive model for HF risk assessment in people with diabetes or prediabetes using ML algorithms using data from the NHANES database with easy‐to‐use clinical and laboratory data, and the RF model we developed can be used by clinicians to make accurate clinical decisions.

Our research does, however, have some drawbacks. First off, because the NHANES database is cross‐sectional in character, the exact order of events is still unknown. Second, the subjects with diabetes or prediabetes we extracted were obtained through patient home interviews. However, the specific subtypes of diabetes or prediabetes are not yet known. Therefore, further studies are needed to investigate the effects of various different types of diabetes or prediabetes on HF. Third, despite the fact that the research first split the entire data set into a training and validation set and used the validation set for internal validation, it still lacks external validation to evaluate the validity of model. Additionally, because our study is based on research done in a U.S. population, a multicenter study with participants from various nations and areas would be necessary to ensure the accuracy of the results. Fourth, the data we used were all extracted from NHANES, and many of the variables were obtained through interviews. This may have impacted the correctness of our data due to potential participant memory biases, which would have impacted the objectivity of the findings.

## 5 CONCLUSION

In this study, we used ML algorithms based on the NHANES database to build five prediction model for the risk of HF in patients with prediabetes or diabetes. The RF algorithm demonstrated the best prediction performance out of the five ML algorithms. The developed RF model is capable of recognizing high‐risk patients in clinical practice and serve as a practical prediction tool for physicians.

## AUTHOR CONTRIBUTIONS

Yicheng Wang and Yan Zhang designed the study. Yicheng Wang processed data, performed statistical analysis and drafted the manuscript. Yan Zhang, Riting Hou, Binghang Ni and Yu Jiang carefully revised the intellectual content.

## CONFLICT OF INTEREST STATEMENT

The authors declare no conflict of interest.

## Supporting information

Figure S1: ROC Curves in the training set.

Figure S2: ROC Curves in the testing set.

Figure S3: Calibration Curves in the training set.

Figure S4: Calibration Curves in the testing set.

Figure S5: DCA Curves in the training set.

Figure S6: DCA Curves in the testing set.

Figure S7: Relative importance ranking of each input variable for Random Forest model.

## Data Availability

The data that support the findings of this study are openly available in National Health and Nutrition Examination at https://www.cdc.gov/nchs/nhanes/index.htm.
