# Supplementary figures and images for: Development and validation of a prediction model based on machine learning algorithms for predicting the risk of heart failure in middle‐aged and older US people with prediabetes or diabetes
Source: Clin Cardiol. 2023 Jul 31;46(10):1234–43. doi: 10.1002/clc.24104 (PMC10577538; doi:10.1002/clc.24104)

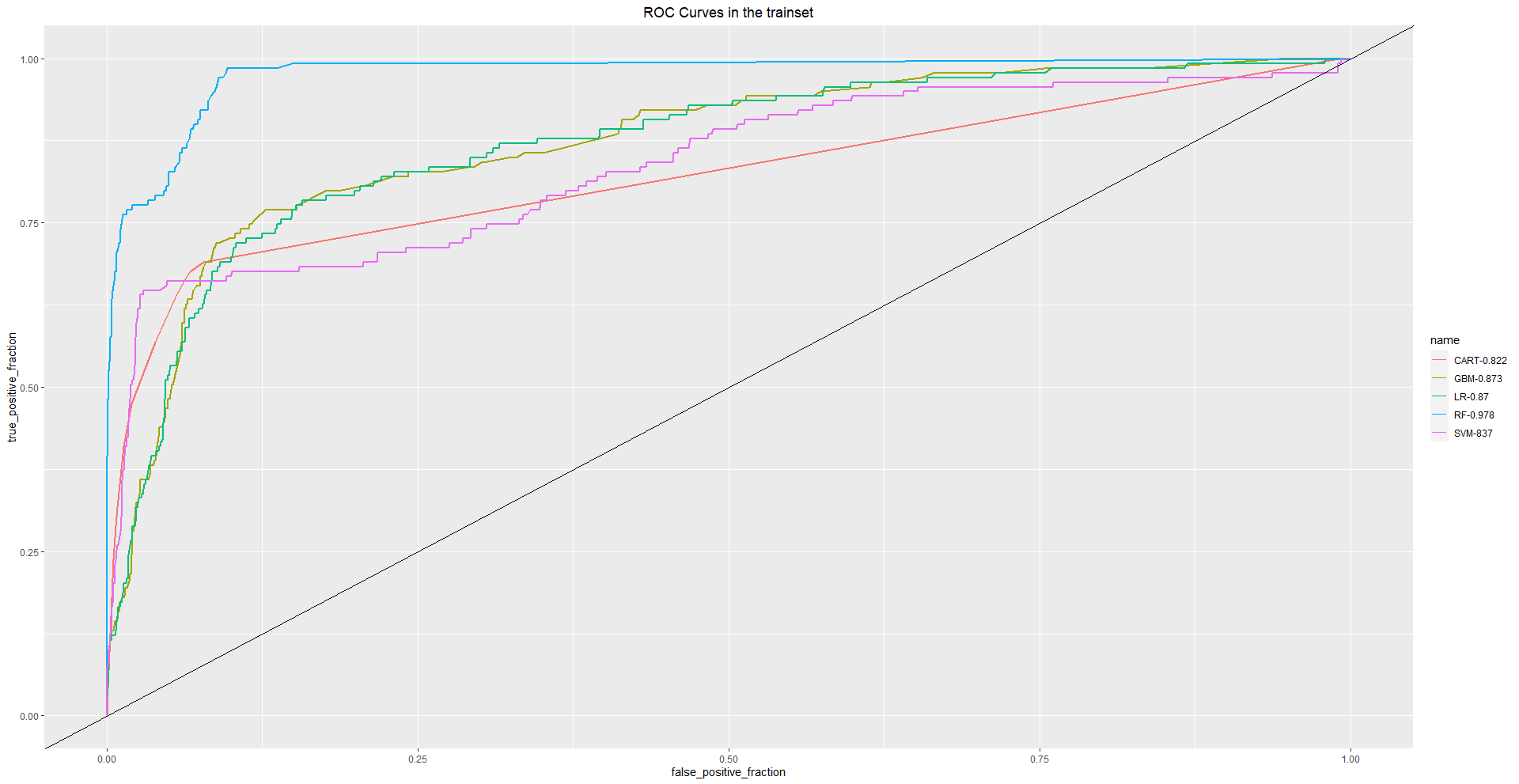

Supplement: Supplementary file 1 — Figure S1: ROC Curves in the training set. [file CLC-46-1234-s001.TIFF]

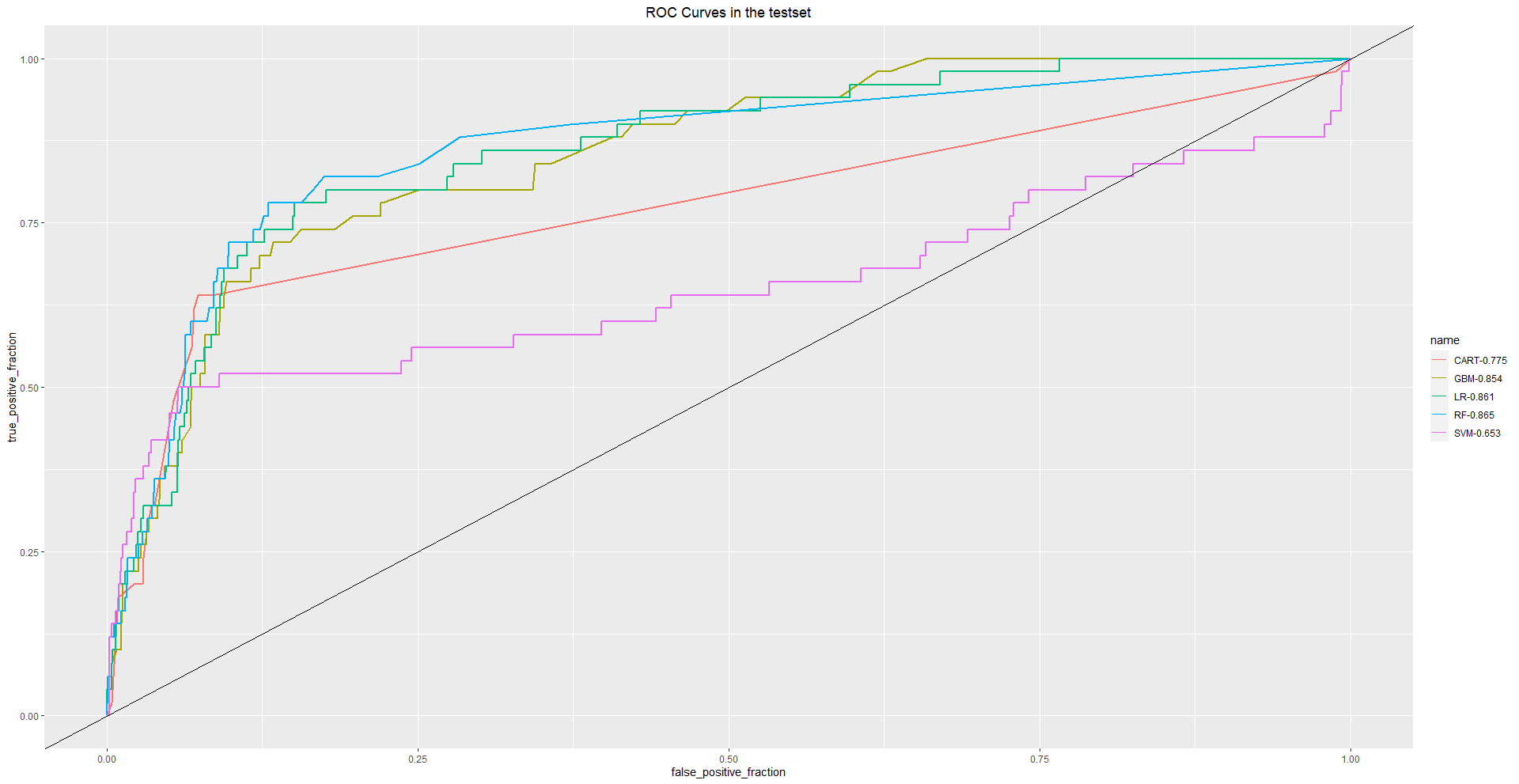

Supplement: Supplementary file 2 — Figure S2: ROC Curves in the testing set. [file CLC-46-1234-s004.TIFF]

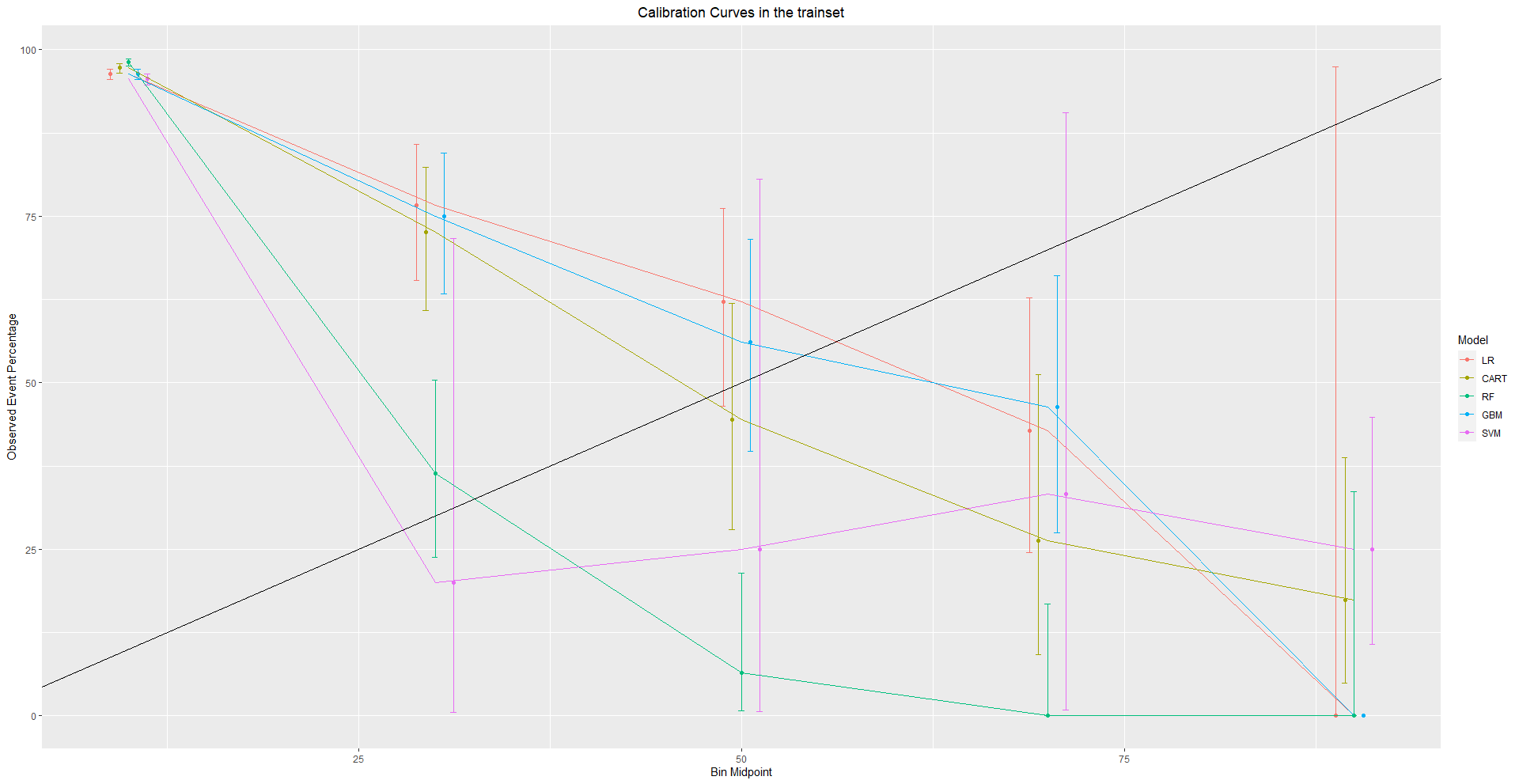

Supplement: Supplementary file 3 — Figure S3: Calibration Curves in the training set. [file CLC-46-1234-s003.TIFF]

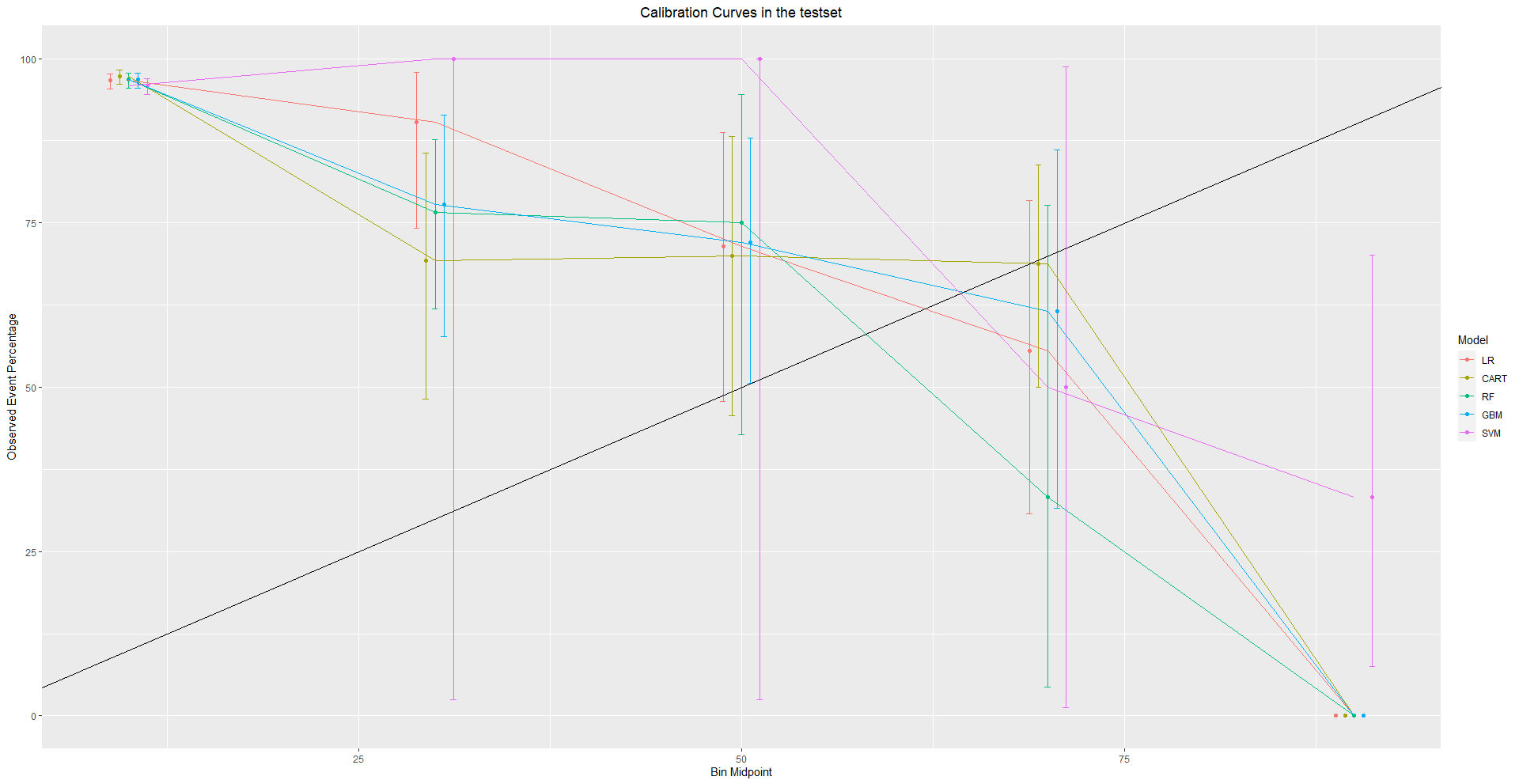

Supplement: Supplementary file 4 — Figure S4: Calibration Curves in the testing set. [file CLC-46-1234-s002.TIFF]

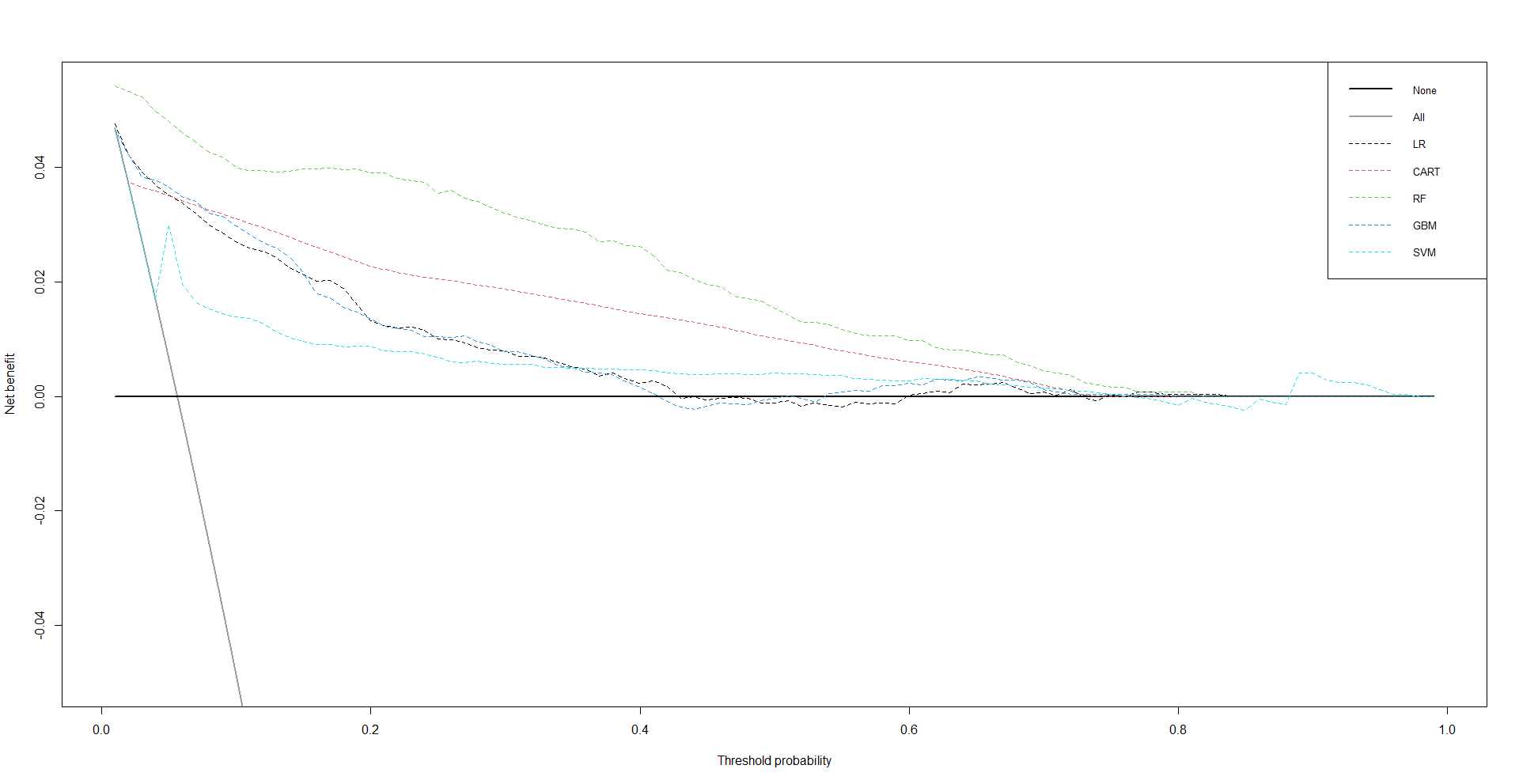

Supplement: Supplementary file 5 — Figure S5: DCA Curves in the training set. [file CLC-46-1234-s006.TIFF]

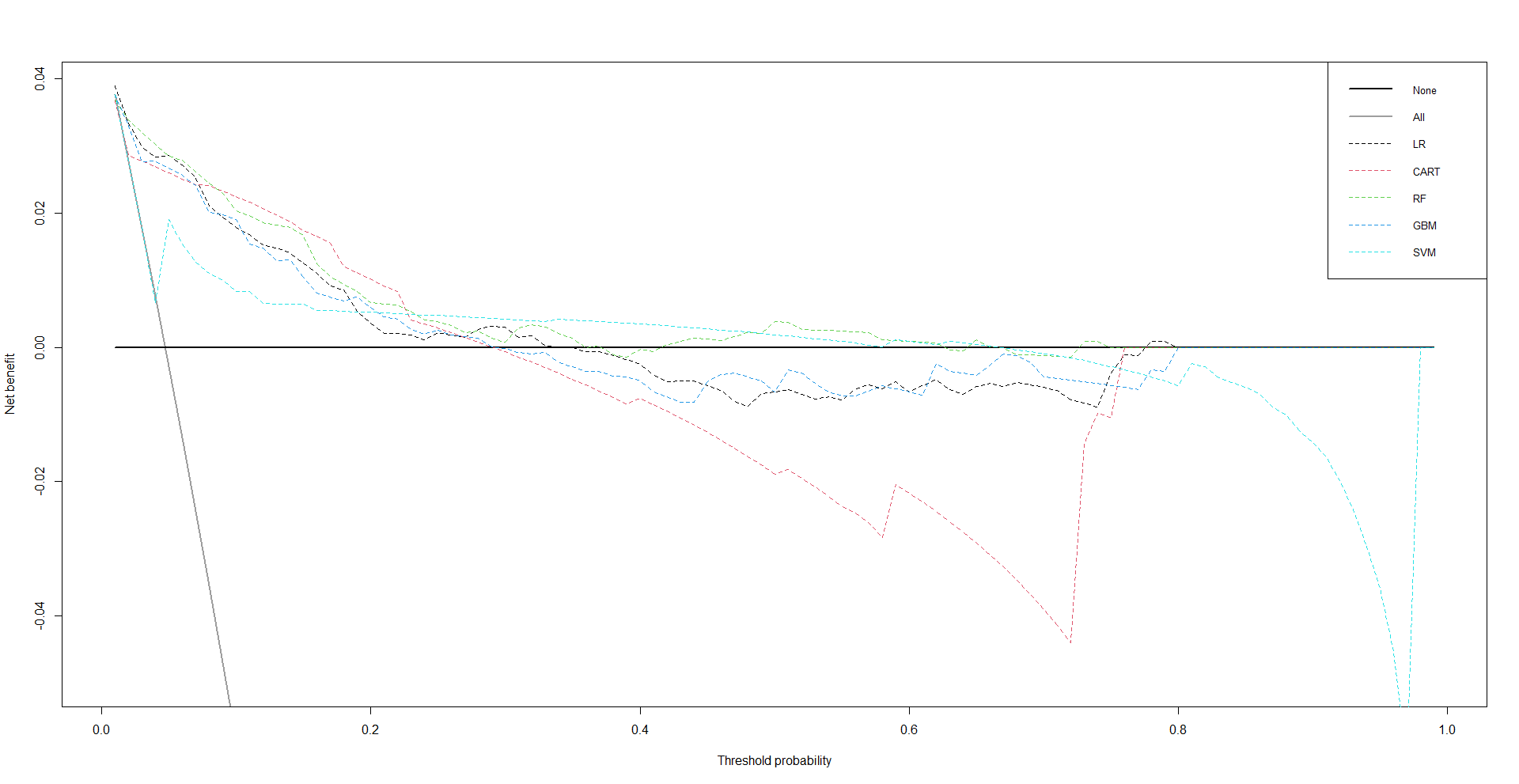

Supplement: Supplementary file 6 — Figure S6: DCA Curves in the testing set. [file CLC-46-1234-s007.TIFF]

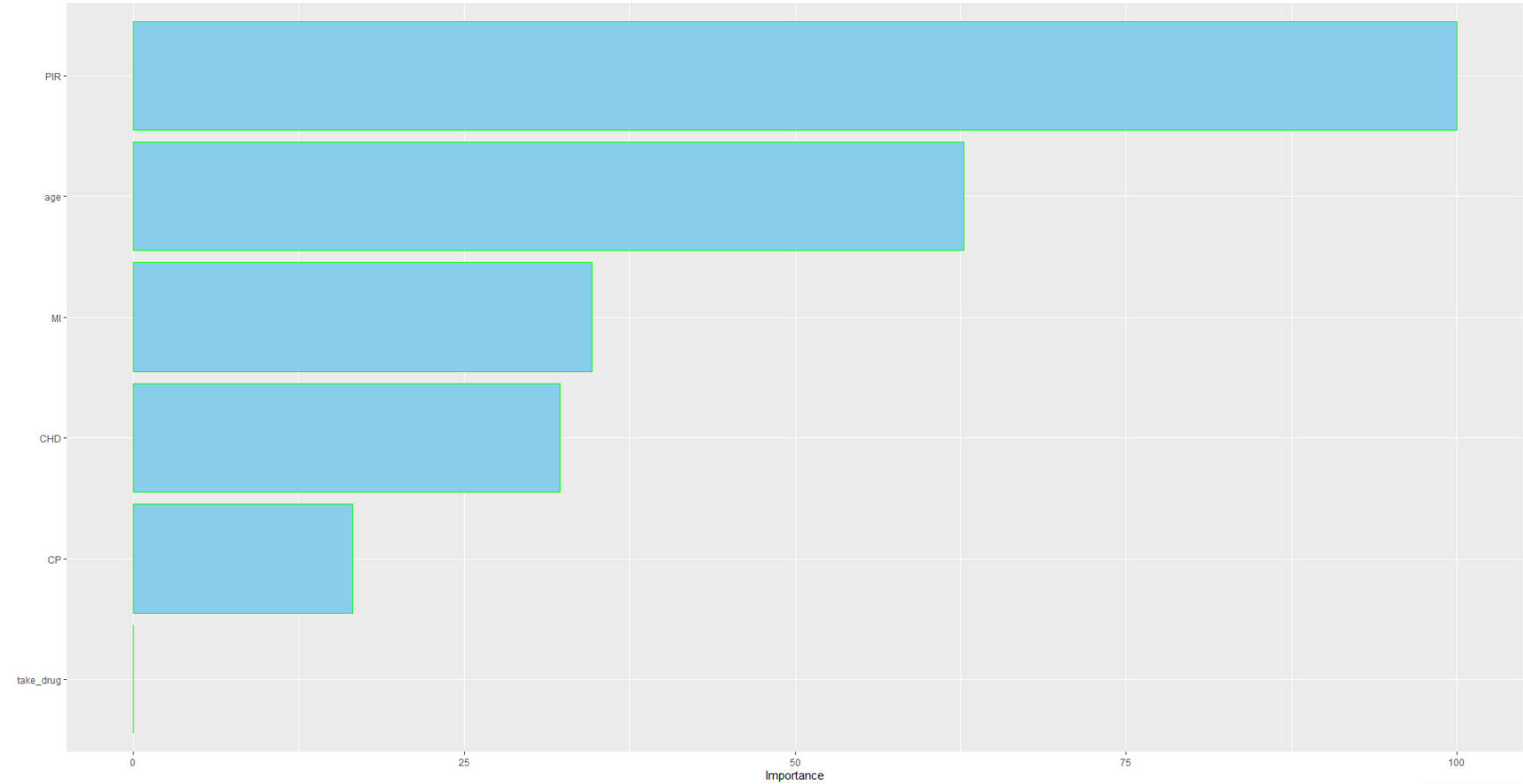

Supplement: Supplementary file 7 — Figure S7: Relative importance ranking of each input variable for Random Forest model. [file CLC-46-1234-s005.TIFF]
